# Scalable lithography from Natural DNA Patterns via polyacrylamide gel

**DOI:** 10.1038/srep17872

**Published:** 2015-12-07

**Authors:** JieHao Qu, XianLiang Hou, WanChao Fan, GuangHui Xi, HongYan Diao, XiangDon Liu

**Affiliations:** 1 State Key Laboratory for Diagnosis and Treatment of Infectious Diseases, Collaborative Innovation Center for Diagnosis and Treatment of Infectious Diseases, The First Affiliated Hospital, School of Medicine, Zhejiang University, Hangzhou, Zhejiang, 310003, China; 2 Key Laboratory of Advanced Textile Materials and Manufacturing Technology, Ministry of Education, College of Materials and Textile, Zhejiang Sci-Tech University, Hangzhou 310018, P.R. China

## Abstract

A facile strategy for fabricating scalable stamps has been developed using cross-linked polyacrylamide gel (PAMG) that controllably and precisely shrinks and swells with water content. Aligned patterns of natural DNA molecules were prepared by evaporative self-assembly on a PMMA substrate, and were transferred to unsaturated polyester resin (UPR) to form a negative replica. The negative was used to pattern the linear structures onto the surface of water-swollen PAMG, and the pattern sizes on the PAMG stamp were customized by adjusting the water content of the PAMG. As a result, consistent reproduction of DNA patterns could be achieved with feature sizes that can be controlled over the range of 40%–200% of the original pattern dimensions. This methodology is novel and may pave a new avenue for manufacturing stamp-based functional nanostructures in a simple and cost-effective manner on a large scale.

Over the past decade, there has been increasing interest in micro-fabrication due to its ubiquitous use in the semiconductor industry, and other new applications, such as micro-fluidic devices, biosensors, tissue engineering, and drug delivery^1^,^2^,^3^,^4^,^5^,^6^,^7^,^8^,^9^,^10^,^11^. Photolithography is the most widely used conventional micro-fabrication technique^12^. However, the intrinsic optical diffraction limitation of photolithography makes patterning features smaller than 100 nm difficult^13^,^14^,^15^. To overcome this challenge, various lithographic approaches, such as electron beam lithography^16^,^17^,^18^, ion beam lithography^19^, and scanning probe lithography^20^, have been developed, but are still cost- and throughput-deficient. Recently, soft lithography^14^,^15^,^21^,^22^,^23^ and nanoimprint lithography^24^,^25^ have attracted attention because of their high throughput and cost efficiency. A suitable stamp is the basic and critical point in both soft lithography and nanoimprint lithography techniques. Stamp fabrication^4^,^7^,^8^,^21^,^26^ can be technologically complicated, and needs specific materials, such as polydimethylsiloxane (PDMS) or quartz. Generally, these stamps have a fixed feature size once fabricated.

Noticeably, component size miniaturization has been a desirable goal in both scientific and industrial fields over the last 25 years^12^,^27^. Particularly in the microelectronics industry, where component size miniaturization often results in faster operation, higher performance, more components per chip, and lower power consumption. Moreover, during the assembly process of complex components, a scalable stamp would bring flexibility to integrating electronic circuitry^28^. polyacrylamide gel (PAMG) is a routine material used in biochemical laboratories. It is hard to think that polyacrylamide (PAM) is better than PDMS in the lithography techniques, because it swells with water, and affecting the patterning regularity. However, with the converse thinking, PAMG may use as a material to prepare a scalable stamp that controllably and precisely shrinks and swells with water content. Moreover, PAM itself is hard and tough in a dry condition.

To fabricate the first master pattern with a desired feature size, self-assembly^29^,^30^,^31^ is an appealing method. The synthetical DNA having well designed sequences has proved to be a versatile building block for creating a wide range of nanostructures^32^,^33^,^34^ (e.g., tile-based arrays^35^, origami^36^,^37^,^38^, complex 2D and 3D structures^39^,^40^) through its superior self-assembling capability. Besides, natural DNA was also applied as an ideal template to fabricate highly ordered nanostructures by binding cationic agents to its negatively charged double helix chains or by its high condensation^41^. Taking DNA nanostructures as study object, the results would provide with representative evidences to the application.

In this study, we conceptually demonstrate a simple yet robust strategy for controllably fabricating precisely sized stamps using patterns created from self-assembled natural DNA. Highly aligned DNA patterns are successfully formed by evaporating aqueous DNA solutions in a curve-on-flat geometry construction situated on a PMMA substrate. A negative replica is obtained by transferring the DNA patterns to unsaturated polyester resin (UPR), and a positive copy is transferred to polyacrylamide gel (PAMG). By drying and swelling of the PAMG stamp, custom size-scalable features are obtained. Furthermore, the shrinking process can be repeated to obtain considerably small structures. Moreover, PAMG stamps are hydrophilic, inexpentive, simply fabricable. They are very feasible for large scale production from various patterns, exspecialy from hydrophilic biopolymers such as DNA, RNA, proten, and carbohydrates. Therefore, this strategy shows promise for application in nanolithography.

## Results and Discussion

### Fabrication of DNA Patterns on PMMA substrate

DNA alignment patterns were successfully prepared using an improved version of a previously reported process^42^. The highly aligned DNA patterns were formed by evaporating natural DNA solution on a smooth, flat PMMA surface in contact with a glass sphere to construct a sphere-on-flat geometry. The directional drying mechanism for DNA alignment is illustrated in SI Fig. 1.

The DNA extremities were specifically bounded to the PMMA surface under a specific pH condition. The DNA is bound strongly enough to withstand capillary forces that occur at the evaporating solution edge (i.e., water/air/PMMA interface) to straighten the DNA molecules. Moreover, the anchored DNA extremities serve as “nucleation sites” for aggregating surrounding DNA molecules into bundles. The glass sphere in contact with the solution surface from above was used to eliminate the temperature gradient and possible convective instabilities caused by water evaporation. Although the salmon milt DNA used for this study has higher molecular weight and wider molecular weight distribution than λ-DNA, highly aligned linear DNA structures were successfully obtained via this improved method.

**Figure 1:**
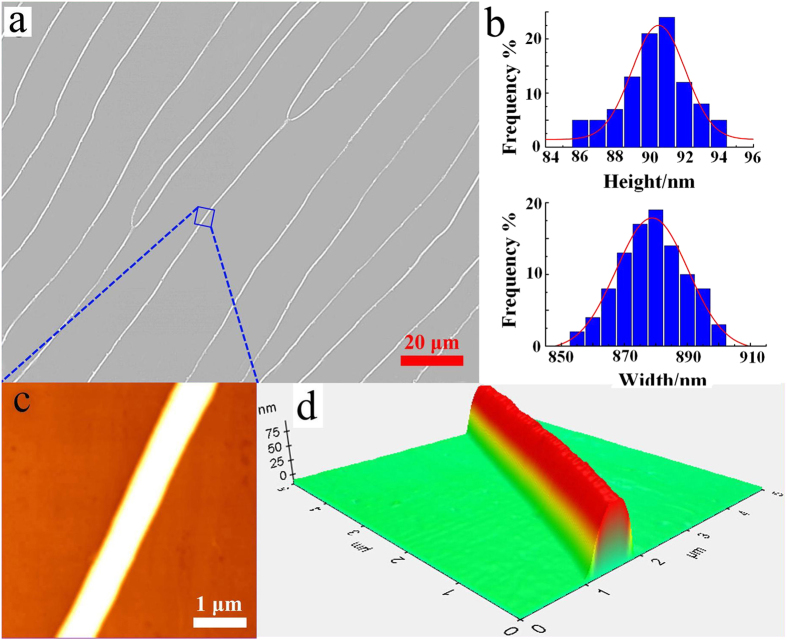
The DNA alignment patterns formed via water evaporation. (**a**) Optical microscopy image of the DNA patterns, and (**b**) their height and width distributions. (**c**) Surface AFM image of the DNA bundle part marked in the optical image a. (**d**) 3D AFM image of the single DNA bundle.

By the optical microscope, it was observed that the DNA alignment patterns have highly aligned regularity on a scale exceeding 1.5 mm (like Fig. 1a). Atomic force microscopy (AFM) images (Fig. 1c,d) reveal that DNA molecules bind together in a straight line on the PMMA surface. The height and width of the linear DNA bundles were statistically calculated from 200 samples measured over more than 30 AFM images. As shown in Fig. 1b, the average height and width of the linear DNA bundles are 90.53 nm and 878.84 nm, respectively, with standard deviation (SD) values of 3.08 and 22.79 nm. The SD values are low, implying that the DNA bundles are uniform over a large region.

### Transfer of the DNA patterns to UPR Surface

**Figure 2:**
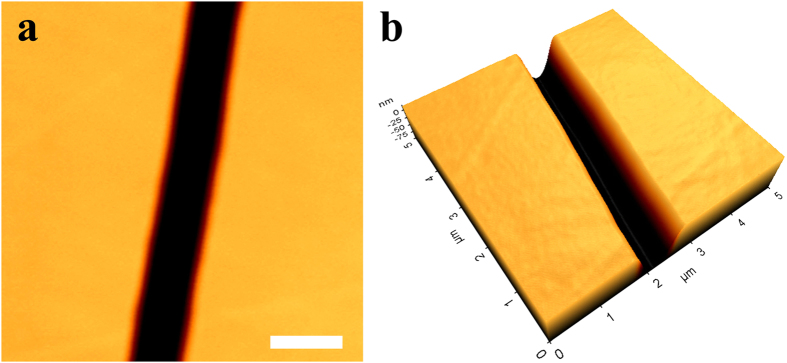
Surface AFM image (a) and 3D AFM image (b) of the UPR negative replica. Scale bar = 1 μm.

DNA patterns cannot be applied as a stamp directly due to the intrinsic chemical instability by hydrolysis^43^, good solubility in water, and weak mechanical strength. Therefore, transferring the DNA patterns to other materials is essential. First, the patterns were transferred to commercially available UPR as a negative replica. The unsaturated polyester was mixed with a curing agent, and outgassed in a vacuum environment. Finally, the DNA patterns were imprinted on the UPR surface by curing the resin. AFM measurements show that the UPR negative replica has a high micro-molding fidelity to the linear DNA patterns, and yields a smooth, flat surface (Fig. 2).

**Figure 3:**
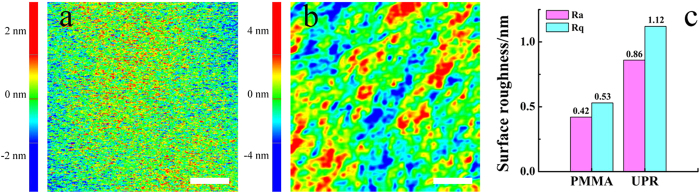
The AFM images of PMMA surface (a) and the UPR surface with the negative patterns replicated from PMMA (b) and the comparison of the surface roughness measured by AFM (c). AFM scanning areas were 5 μm×5 μm. Scale bars = 1 μm.

Although both the average roughness (Ra) and the root mean square roughness (Rq) of the UPR surface are greater than that of the original PMMA surface (Fig. 3c), their values are less than 1.5% of the height of linear DNA patterns (about 90.5 nm), meaning that the UPR surface would not seriously affect the fidelity to the linear DNA patterns (Fig. 3).

**Figure 4:**
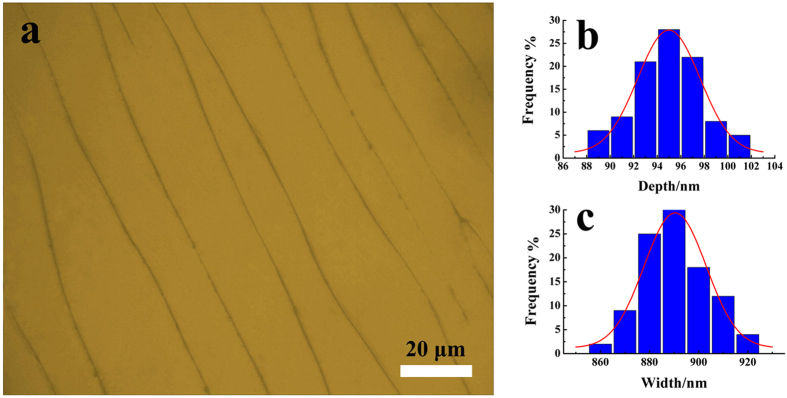
The optical microscopy image of the surface of the UPR negative replica (a) and the depth (b) and width (c) distributions of the linear pattern structures.

Figure 4a presents an optical microscopy image of the UPR negative replica. The statistic data from the AFM images are shown in Fig. 4b,c. The average depth and width are 94.99 ± 5.31 nm and 890.42 ± 25.25 nm, and are in good agreement with the original DNA patterns (Table 1).

### Scalability of the PAMG Stamps

The monomer solution was poured onto the surface of UPR negative replica, and subsequently outgassed in vacuum to remove bubbles at the interface between the pattern and the polymer solution. Next, n-hexane was added to isolate oxygen before the induction period. Finally, the monomer solution was polymerized to form the PAMG stamps with the patterns derived from the linear DNA structures. The PAMG stamps with reduced feature sizes were fabricated by removing the water in the gel.

**Figure 5:**
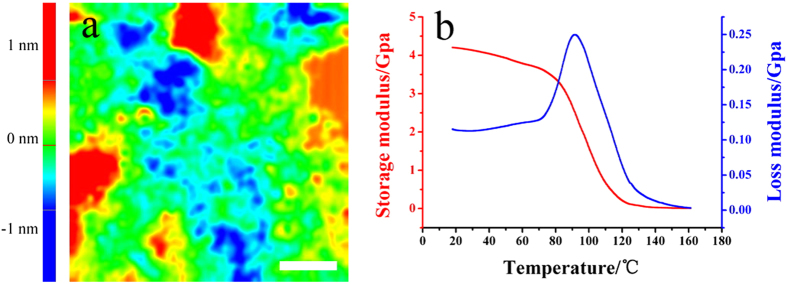
The AFM surface image (a) and DMA spectra (b) of the PAMG stamp. Scale bar = 1 μm.

Figure 5a reveals that Ra and Rq of the shrank PAMG stamp are 0.45 nm and 0.55 nm, respectively. These values are similar to those of PMMA, as shown in Fig. 3 (Ra = 0.42 nm, Rq = 0.53 nm), and have reduced approximately two-fold compared with UPR, demonstrating that the PAMG surface roughness reduces as the feature size is reduced. DMA experiments were performed to evaluate the contracted PAMG stamp. The Dynamic mechanical analysis (DMA) curves in Fig. 5b indicate that the glass transition temperature (*T*_*g*_) of the PAMG stamp is approximately 90 °C, as defined by the loss modulus peak. The PAMG stamp also exhibits a high storage modulus, ranging 2.5–4.2 GPa at room temperature. Both of these properties meet the standards for daily use of the stamp without deformation.

**Figure 6:**
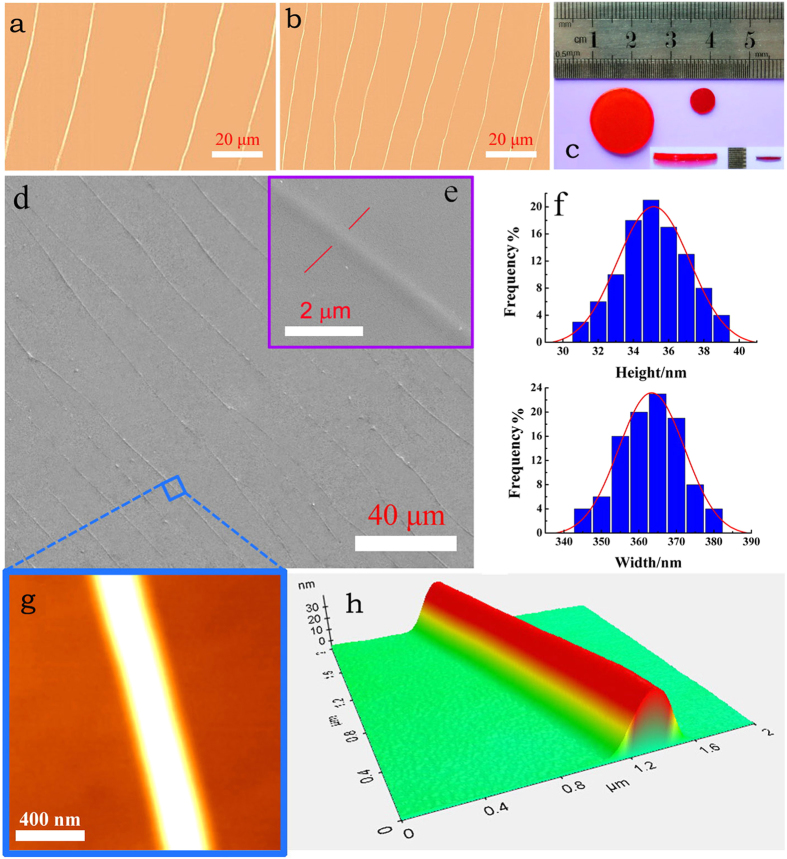
The alignment patterns on the shrank PAMG stamps. (**a**) Optical microscopy image of the PAMG patterns without size reduction. (**b**) Optical microscopy image of the shrank PAMG patterns. (**c**) Photograph of the PAMG stamps before (left) and after (right) shrinking. (**d,e**) SEM images of the shrank PAMG stamp surface. (**f**) Height and width distributions of the contracted linear structures. (g and h) AFM images of the linear structure on the shrank PAMG surface.

The pattern shrinkage of the PAMG stamp was examined with optical microscopy (Fig. 6a-c), and no distortion or cracking were observed. The SEM images (Fig. 6d,e) show that the patterns appeared on the PAMG stamp surface are similar to the original DNA structures. Basing on more than 30 AFM images like Fig. 6g,h, the height and width of the features were statistically calculated (Fig. 6f). Their average vaules are 35.15 ± 4.11 nm and 363.38 ± 17.66 nm, respectively, indicating that the feature sizes of the PAMG stamp are about 2.5 times smaller than (or about 40% to) the UPR negative replica or original master (Table 1).

**Figure 7:**
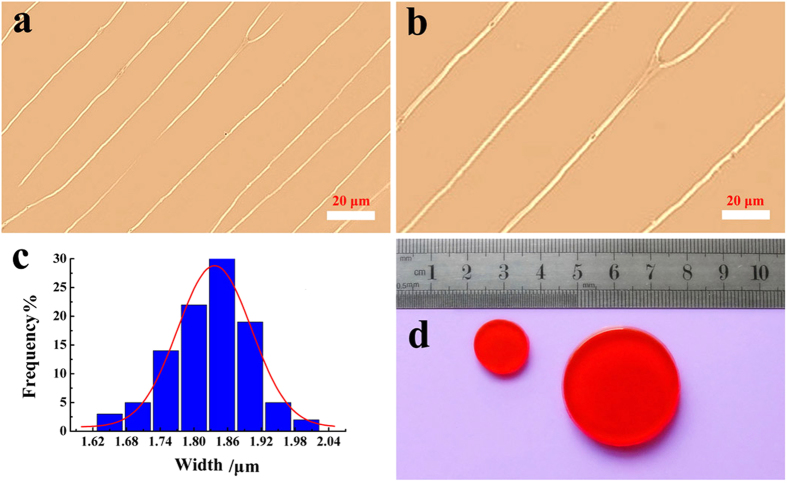
The alignment patterns on the enlarged PAMG stamp. (**a**) Optical microscopy image of the PAMG pattern without size enlargement. (**b**) Optical microscopy image of the enlarged PAMG patterns. (**c**) Width distribution of enlarged linear structures. (**d**) Photograph of the PAMG stamps before (left) and after (right) enlarging treatment.

PAMG may also be utilized for fabricating enlarged stamp via water absorption, as shown in Fig. 7a,b. An average width of 1.84 ± 0.13 μm is statistically calculated from the optical images for enlarged linear structures (Fig. 7c). The width of the linear PAMG structures can be swollen up to 2 times than the original DNA patterns. This microscopic swelling result of the enlarged linear structures was consistent with the macroscopic photograph of the swelled gel samples shown in Fig. 7d.

**Figure 8:**
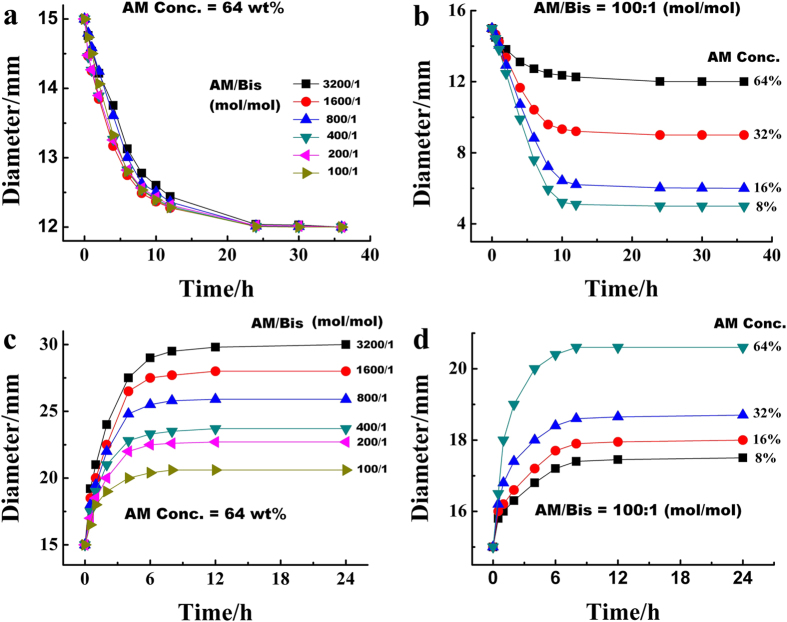
The tunableness of the PAMG by evaporation or swalling in water. The diameter of the PAMG stamps as a function of evaporation time (**a,b**) and swelling time (**c,d**) respectively. Conditions for fabrication of the PAMG stamps: a fixed AM concentration (64 wt%) with a varity of AM/Bis molar ratios was used for samples a and c, a fixed AM/Bis molar ratios (100:1) with a varity of AM concentrations for samples (**b,d**).

To implement the size controllability of the PAMG stamp, the effects of various parameters, such as the Acrylamide (AM): Bis-acrylamide (Bis) molar ratio and the concentration of AM monomer, were studied. As shown in Fig. 8a, the diameter reductions of the PAMG stamps as a function of time were comparable for all AM: Bis molar ratios. However, when the molar ratio of AM: Bis is 100:1, the final diameter reaches values of to 12 mm, 9 mm, 6 mm, and 5 mm as the AM concentration is reduced from 64% to 8% (Fig. 8b). Therefore, the AM monomer concentration plays a key role in the diameter reduction. In contrast, both the AM: Bis molar ratio and the AM monomer concentration strongly influence the size of the PAMG stamp in the enlarging experiments. As shown in Fig. 8c,d, the decrease of Bis concentration and the increase of AM concentration can result in a larger diameter of the PAMG stamp after an enlargement treatment by adsorbing water. As the concentration of Bis increases, the PAMG network becomes denser and simultaneously the free space within the polymeric matrix that favors water absorption is reduced. Similarly, along with the increase of AM concentration, diameter increases due to the increase in water absorbency in the PAMG system. Utilizing the relationship between diameter and evaporation or absorption time, size-scalable stamps could be easily fabricated by adjusting polymer monomer ratio and concentration of AM monomer.

In summary, a simple yet robust strategy for fabricating scalable patterns is demonstrated by copying natural DNA patterns that were self-assembled linearly with the assistance of a curve-on-flat geometry. The patterns were first transferred to a UPR negative replica, and then copied to a PAMG positive. By controlling the drying and swelling of the PAMG stamp, scalable patterns with customized feature sizes were obtained. The feature sizes of the patterns on the PAMG stamp can be tailored by adjusting the water content in the PAMG, and the achievable range of the pattern feature size is 40% to 200%. It is possible to produce considerably smaller structures by repeating the size reduction process. This facile strategy may provide a new avenue for nanolithography techniques for achieving pattern scalability, and is applicable on massive fabrication scales in a simple and cost-effective manner.

## Experimental Section

### Materials

Salmon milt DNA (6.6 × 10^3^ kDa, 10 Kbp; melting temperature, 84 °C; UV absorbance ratio of A260 nm/A280 nm, 1.84) used in this work was purchased from Yuki Gosei Kogyo Co., LTD. (Fukushima, Japan). The UPR was an orthophthalic polyester resin obtained from Jinling DSM Resins Co., Ltd. (Nanjing, China). All other reagents were purchased from Aladdin Industrial Co., Ltd. (Shanghai, China) and without further purification. Ultrapure water with a resistivity of 18.2 MΩ•cm was used in all experiments.

### Instruments

Surface morphology was investigated by utilizing JSM-6700F filed emission scanning electron microscope (FE-SEM, JEOL, Japan) after platinum coating (10 nm thickness). AFM images were obtained from an AFM instrument (PSIA XE-100E, Suwon, Korea) using tapping mode. DMA was performed with a TA Instruments Q800 DMA (USA) with a heating rate of 5 °C /min from 25 °C to 160 °C at 1.0 Hz.

### Preparation of Natural DNA Buffer Solution

Briefly, EDTA-2Na (0.58 g, 1.57 mmol) and Trisodium citrate dehydrate (0.59 g, 2.00 mmol) were added to 200 ml of water, stirred for 30 min, and added with 1.00 M HCl solution to adjust the pH to 6.20. Salmon milt DNA (500 μg/ml in 1 mM EDTA/10 mM Tris•HCl, pH = 8.00) was directly added into the buffer solution described above to yield 10 μg/ml Salmon milt DNA solution.

### Fabrication of DNA Patterns on PMMA substrate

A curve-on-flat geometry was constructed by a glass sphere (10 mm diameter) on a flat PMMA substrate (15.0 mm ×15.0 mm ×2.00 mm). A droplet (45.0 μL) of the DNA buffer solution was dropped on the centre of the PMMA substrate, and the glass bead was put upon the droplet gently. The entire device was heated in an oven at 65 °C overnight to obtain the patterns^42^.

### Transfer of the DNA patterns to UPR Surface

A mixture of UPR (3.0 ml), benzoyl peroxide (initiator, 0.1 mmol) and N,N,4-trimethylaniline (promoter, 0.03mmol) was poured on the original master (i.e., the DNA patterns on PMMA surface) and deaerated in vacuum. After cured for 2 min, the UPR negative replica was peeled off gently, and analyzed using AFM measurement.

### Fabrication of PAMG Stamps

Monomer solution (5 ml) of AM and Bis with a certain mole ratio (AM:Bis = 400:1 and 3200:1 were used for shrinking and enlarging PAMG stamps, respectively) was mixed with 40 μl of Ammonium persulfate aqueous solution (10 wt%) and 5 μl of N,N,N’,N’-Tetramethylethylenediamine (TEMED), poured to the UPR negative replica, deaerated in vacuum for 2 min, added n-hexane (2 ml) to isolate oxygen, and polymerized in 25 °C to obtain PAMG. The stamp was peeled off carefully and dried at 65 °C overnight.

## Additional Information

**How to cite this article**: Qu, J.H. *et al.* Scalable lithography from Natural DNA Patterns via polyacrylamide gel. *Sci. Rep.* **5**, 17872; doi: 10.1038/srep17872 (2015).

## Supplementary Material

Supplementary Information

## Figures and Tables

**Table 1:** The feature size of the patterns in each step^a^

Pattern	DNA Alignment	UPR negative replica	Shrank PAMG Stamp
Width (nm)	878.84 ± 22.79	890.42 ± 25.25	363.38 ± 17.66
Height (nm)	90.53 ± 3.08	/	35.15 ± 4.11
Depth (nm)	/	94.99 ± 5.31	/

^a^statistic data from multiple AFM images.
