# Supplementary material for: Scalable lithography from Natural DNA Patterns via polyacrylamide gel
Source: Sci Rep. 2015 Dec 7;5:17872. doi: 10.1038/srep17872 (PMC4671135; doi:10.1038/srep17872)
Supplement: Supplementary Information [file srep17872-s1.pdf]

Supplementary Information

Scalable lithography from Natural DNA Patterns via polyacrylamide gel

JieHao Qu,[ab] XianLiang Hou,[a] WanChao Fan,[b] GuangHui Xi,[b] HongYan Diao,[a] and XiangDong Liu[b]

[a.] State Key Laboratory for Diagnosis and Treatment of Infectious Diseases, Collaborative Innovation Center for Diagnosis and Treatment of Infectious Diseases, The First Affiliated Hospital, School of Medicine, Zhejiang University, Hangzhou, Zhejiang, 310003,China.

[b.] Key Laboratory of Advanced Textile Materials and Manufacturing Technology, Ministry of Education, College of Materials and Textile, Zhejiang Sci-Tech University, Hangzhou 310018, P.R. China.

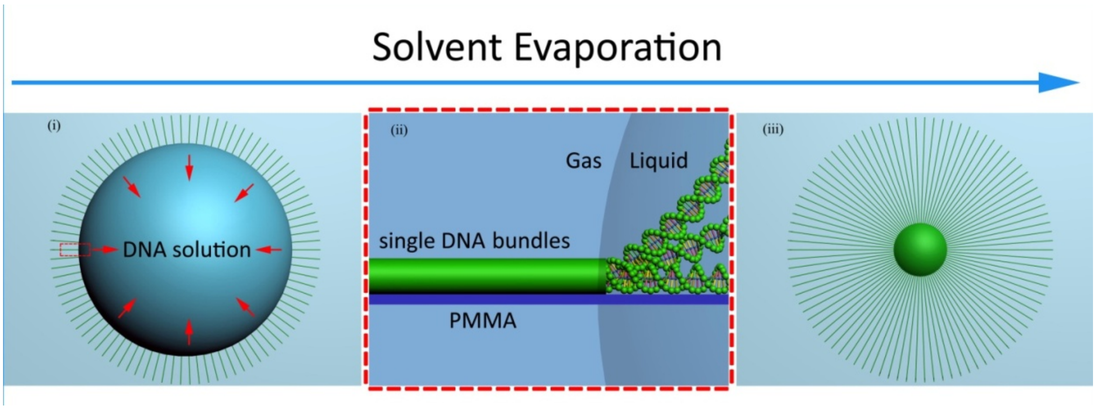

SI Figure 1 (i) Schematic illustration of the evaporating DNA solution droplet with the developping DNA bundles, (ii) at the water/air/PMMA interface, and (ii) the resulting DNA bundles under the glass bead.
